# Supplementary material for: Chromothripsis during telomere crisis is independent of NHEJ, and consistent with a replicative origin
Source: Genome Res. 2019 May;29(5):737–49. doi: 10.1101/gr.240705.118 (PMC6499312; doi:10.1101/gr.240705.118)
Supplement: Supplemental Material [file supp_gr.240705.118_Supplemental_file_1.zip › contigs/annotated_contigs/DB46/contig.2.DB46_length_992_mean_cov_9.22379032258.docx]

**DB46_length_992_mean_cov_9.22379032258**

```
AAAAATGCGTGGCTTTATGCGTGAGCTTAGTTCTTAGCTTTATACACACAATATACATATTTCTATTTTGGGTAAAGTTAAAATAACCA
 >chr18:57656441-57657019 + E=0e+00
AAATAATTAATTATTCTGGCCTTTTTATGTGGCCATTTGACTCTTTTAAGTGTGTTAAAATATTTGCTTTACAAGTAGCTGGTCTTTGC

TTATGTTTAGATAAGTACCTGTGTTATTGCCCTGTTCATTTAGTAATCTAAAATTCTAAGCCAGGGGAGCCATCACCAGGTGATATGGT

TTGGCTCGGTGTGCCCACCCAAATCTCATCTTGAATTATAATCCCCACATGTCAAAGGAGGGGCCTGGTGGGAGAGATGATTGAAAAAT

GGGGTTGGATTTCTCCCTTGCTGTTCTCATGATAGTGAGTGAATTCTCACAAGCGTTGATGGTGCTTCCCCCTTCGTGTACACTCTCTC

TCTCCTGCTGCCATGTAAGGTGTGCCTTGCCTCCCATTCCATCATCACTGTAAGTTTCCTGAGGCCTCCCCAGCCATATGGGACTGTGA

GTCAACTAAACCTTCTTTTTATAAATTACCCAGTCTCAGGTAG|T|GCCATTGCACTCTAGCCTGGGCGACAAGAATGAAACTCCATCT
                                            >chr18:57642994-57643371 + E=9e-209
CGGACGGGGGCGGGCAGGTGAAGAGTGCAGAGAACAATGTTTTCATTTTATAAGGCTTGTTTCTAGGACATTTTGGAATTCTTTGCTAC

TATTCCTTTGATTCTAAAATTTTTGATAATGAAGTGTTTGGATAGAACAGATGTATTGACATCAAAAATATTATTAATGTTTACTCTAA

TTTTAGAATAATTCATGTTTATTAAACACCAAAGGGATTTCCTTTCTTTTGAAATTATTTCAGATATACAAAGTACAAAAATAAGATAA

TAAACACTCATGTACCACTGCCCAGATCTTACAAATGACATTGTTGCTACATATTGGCTCAGATTT|CGGAGACAAGAAATAATACATG

CGTCAGAGTTGATCGG
```
